# *STXBP1*-Related Developmental and Epileptic Encephalopathy

**DOI:** 10.15844/pedneurbriefs-33-6

**Published:** 2019-12-31

**Authors:** Brittani Wild, Stephen Nelson

**Affiliations:** 1 Departments of Pediatrics and Neurology, Tulane University School of Medicine, New Orleans, LA

**Keywords:** STXBP1, Epilepsy, Intellectual disability, Early-Onset Epileptic Encephalopathy

## Abstract

Researchers from the University of Antwerp, Belgium, and numerous international collaborators report a comprehensive overview of the phenotypic and genetic spectrum of Syntaxin-binding protein 1 (*STXBP1*) encephalopathy.

Researchers from the University of Antwerp, Belgium, and numerous international collaborators report a comprehensive overview of the phenotypic and genetic spectrum of Syntaxin-binding protein 1 (*STXBP1*) encephalopathy. The article provides a phenotypic summary of 147 patients with *STXBP1*-E pathogenic variants, including 45 previously unreported patients with 33 novel *STXBP1* variants. Researchers used statistical analysis to further classify variant type (missense vs truncating) and cognitive outcome (mild to moderate intellectual disability, vs severe to profound) and cognitive outcome and seizure outcome (seizure-free vs not seizure-free). Age of inclusion criteria ranged 6 months to 56 years with a mean of 5.75 years. At the onset of seizures, 53% of patients were diagnosed with EOEE, 20% with Ohtahara Syndrome (most with evolution to West Syndrome), 9% with West Syndrome, 6% with intellectual disability with non-syndromic epilepsy, and 2% with Dravet Syndrome. One additional patient was reported to have early myoclonic encephalopathy and one patient reported to have intellectual disability with two possible seizures.

Regarding epilepsy associated with *STXBP1* pathogenic variants, 95% of patients were reported with epilepsy with most frequent seizure types consisting of epileptic spasms (65.3%), focal seizures (57.9%), and tonic seizures (41.3%). Seizure freedom was attained in 1 in 3 patients while 1 in 3 remain therapy resistant. Intellectual disability was found in all patients, with 88% of these patients classified in the severe to profound range. Greater than 60% of patients had focal or multi-focal epileptic activity on EEG. Other common EEG findings were burst suppression (35%) and hypsarhythmia (40%). Behavioral problems associated with Autism or autistic features was seen in 1 in 5 patients. Other common phenotypic symptoms include axial hypotonia, ataxia or ataxic gait, tremor, spasticity and dyskinesia, or dystonia. MRI findings were normal in almost half, with abnormal findings consistent with cerebral atrophy (33%), thinning of the corpus callosum (16%), or hypomyelination (16%).

There was a higher incidence of severe to profound intellectual disability associated with missense variants (91%) compared to truncating variants (86%). Out of the 9 patients without epilepsy, 6 carried truncating variants and 3 carried missense. The authors noted that *STXBP1* should furthermore be classified as a complex neurodevelopmental disorder vs primary epileptic encephalopathy due to the high incidence of intellectual disability associated with *STXBP1* with little correspondence to age of seizure onset or severity. [1]

COMMENTARY. This paper provides a comprehensive phenotypic and genetic analysis of individuals with *STXBP1* pathogenic variants. Although most patients with *STXBP1*-related disease present with epilepsy, others may have primarily movement disorders such as an ataxia-tremor-retardation syndrome [2]. In some selected patients with drug resistant epilepsy, surgical intervention has been reported to successfully reduce seizure frequency [2,3]. Although profound intellectual disability is highly associated with *STXBP1* variants, autism spectrum disorder is rarely seen [3]. Management typically includes anticonvulsants for seizure control and early intervention with physiotherapy, occupational, speech and or behavioral therapy to treat the complex neurodevelopmental aspects of the disorder [1,3].

## Disclosures

The authors have declared that no competing interests exist.
